# Effects of Process Parameters on Material Removal in Vibration-Assisted Polishing of Micro-Optic Mold

**DOI:** 10.3390/mi9070349

**Published:** 2018-07-12

**Authors:** Jiang Guo, Hirofumi Suzuki

**Affiliations:** 1 Key Laboratory for Precision and Non-Traditional Machining Technology of Ministry of Education, Dalian University of Technology, Dalian 116024, China; 2 Department of Mechanical Engineering, Chubu University, 1200 Matsumoto-cho, Kasugai, Aichi 487-8501, Japan; suzuki@isc.chubu.ac.jp

**Keywords:** polishing pressure, vibration, material removal, roughness, tungsten carbide, tool wear

## Abstract

Process parameter conditions such as vibrating motion, abrasives, pressure and tool wear play an important role in vibration-assisted polishing of micro-optic molds as they strongly affect material removal efficiency and stability. This paper presents an analytical and experimental investigation on the effects of process parameters, aimed at clarifying interrelations between material removal and process parameters which affect polishing quantitatively. The material removal rate (MRR) and surface roughness which represent the polishing characteristics were examined under different vibrating motions, grain sizes of abrasives and polishing pressure. The effects of pressure and tool wear conditions on tool influence function were analyzed. The results showed that 2D vibrating motion generated better surface roughness with higher material removal efficiency while a smaller grain size of abrasives created better surface roughness but lower material removal efficiency. MRR gradually decreases with the increase of polishing pressure when it exceeds 345 kPa, and it was greatly affected by the wear of polisher when wear diameter on the polisher’s head exceeds 300 μm.

## 1. Introduction

There is an increasing demand for micro-optic lenses with low-cost and high optical qualities in consumer digital optical devices such as digital cameras, DVD players and microscopes. Besides that, micro-optic lenses are widely used in medical and industrial applications where their small size and renowned high-quality optical imaging are important [1,2]. Due to the large number of requirements and the complexity in structure, the molding process becomes a highly productive method for providing high accuracy and shape control of the manufactured parts. To withstand high temperature and pressure conditions in the hot-press molding process for replicating glass lenses, tungsten carbide (WC) material is usually adopted [3,4]. Due to its hard-brittle nature, a grinding process is required for generating the form. Some research has been done on grinding methods and tools to achieve high form accuracy [5,6,7,8]. However, a super-smooth surface in high form accuracy is still difficult to obtain in order to meet the requirements well. Therefore, a subsequent polishing process with loose super abrasives becomes necessary.

Several super-fine polishing technologies such as magnetorheological finishing (MRF), fluid jet polishing, and bonnet polishing, have been developed for finishing complex optics in recent years, but they are still limited to relatively large-size optics [9,10,11,12,13]. As an alternative technology, vibration-assisted machining shows strong capabilities to machine small-size parts [14,15,16]. To polish small-size optics, recently, a vibration-assisted polishing method has been proposed and successfully applied to finish the micro-optic molds with high form accuracy and low surface roughness [17,18,19,20]. Compared with the polishing methods using rotation, it has the advantages that (1) owing to the two-dimensional (2D) vibration with shorter polishing traces, better surface roughness can be obtained; (2) the polishing tool can be focused on polishing a very small area (under 0.2 mm^2^) with high removal efficiency; (3) since polishing efficiency is dominated by vibration frequency and amplitude, not by the polishing tool’s diameter, it can be miniaturized and arbitrarily shaped to meet specific requirements. So, vibration-assisted polishing is quite suitable to be applied to polish micro-optic molds of diameter less than 5 mm with a concave shape and large curvature. To control the polishing process well, the effects of process parameters in the material removal process needs to be well understood because it is crucial to ensuring high process repeatability. However, polishing is a very complex phenomenon which is affected by many variables. Due to the interactions between polisher, workpiece and abrasives and the physical scale of the material removal processes in polishing being difficult (practically impossible) to observe directly [21,22], up to now, the fundamental material removal mechanism is still poorly understood and a holistic knowledge of it does not exist although during the last decades efforts have been made to investigate interrelations between process parameters and removal mechanism [23].

This paper presents an analytical and experimental investigation on the effects of process parameters, aiming at clarifying interrelations between material removal mechanism and parameters which affect the polishing process qualitatively. Firstly, the effects of process parameters including different vibrating motions of the polisher and grain sizes of abrasives on material removal rate (MRR) and surface roughness are examined. Then the effects of pressure and tool wear on tool influence function are analyzed. To reveal some insights of the mechanism involved in vibration-assisted polishing, a general model of material removal is proposed to illustrate the phenomenon of MRR change by varying polishing pressure. Finally, as another parameter which greatly affects MRR, the wear of the polisher is discussed with some experimental verification.

## 2. Experimental Setup and Conditions

**Figure 1:**
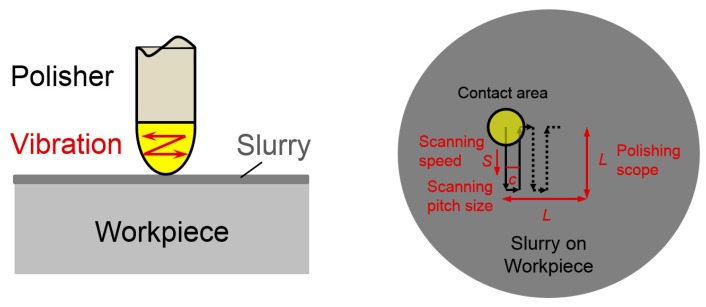
The scanning path of polisher on workpiece surface.

The experiments were conducted using similar setups as previous research. The main polishing conditions are summarized as shown in Table 1. Tungsten carbide was adopted as the workpiece material since it is a hard-brittle material which can withstand high temperature and pressure conditions in the hot-press molding process to replicate the glass lenses. It was lapped prior to polishing on a lapping machine. A polisher made of polyurethane was selected for the experiments. It is a kind of soft but hard-to-wear material with an international rubber hardness degree (IRHD) of 90. The shape of the polisher was generated by precision cutting process with high form accuracy. It can be miniaturized with a radius of curvature from 0.5 mm to 2 mm. In this paper, a radius of 1 mm is used. Concerning surface roughness and material removal efficiency, diamond slurry is the best abrasive to be used to polish tungsten carbide, so a few kinds of diamond slurry with density of 1 wt % and grain size of 0.1 μm, 0.125 μm, 0.25 μm, 0.5 μm and 1 μm were employed for testing. These diamond slurries are water-based with excellent cutting performance and surface finish and they are particularly effective for materials which are hard to machine. Since loading conditions appear to be key to understanding the removal mechanism [24], this paper will focus on them. As mentioned above, the polishing force can be precisely controlled by the real-time polishing force control system, so it is set from 5–50 mN with an increment of 5 mN in the experiments. The magnetostrictive vibrating polisher vibrated at frequency of 9.2 kHz. Lateral, circular and elliptical vibration traces correspond to a phase difference of 0 deg, 45 deg and 90 deg, respectively, in the opposing pair of coils of magnetostrictive vibrating polisher [19]. The vibration amplitude in lateral vibrating motion was 43 μm. The radius of circular vibrating motion was 30 μm. The long axis and minor axis of elliptical vibration was 40 μm and 16 μm, respectively. For the lateral vibration motion, the vibration direction is along the scanning path. For the experiments to investigate interrelations between material removal and process parameters, the raster scanning path was adopted. As shown in Figure 1, the polishing tool vibrated on a workpiece surface during scanning and the material was removed in a rectangular shape. The polishing scope was set to 400 μm × 400 μm, while the polishing tool had a scanning speed of 3.5 mm/min. with a pitch size of 20 μm.

## 3. Analysis of Pressure and Tool Wear Effects

**Figure 2:**
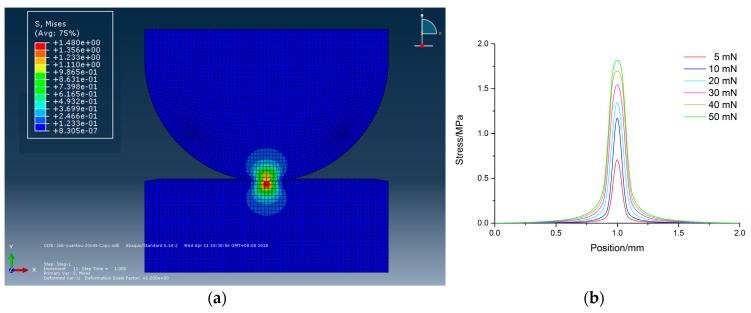
Stress distribution w/o tool wear (**a**) cross-sectional view at 20 mN, and (**b**) under different loads on workpiece surface.

**Figure 3:**
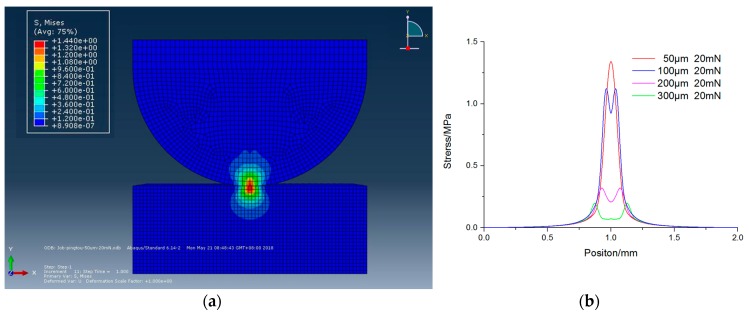
Stress distribution with tool wear at 20 mN (**a**) cross-sectional view with wear diameter of 100 µm, and (**b**) with wear different diameters on workpiece surface.

The effects of pressure and tool wear conditions on tool influence function were analyzed to know the stress distributions. A finite element model (FEM) was developed based on contact mechanics with the commercial FEM software ABAQUS (ABAQUS6.14, Dassasult Simulia Company, Providence, RI, USA). The static contact model was built considering the boundary conditions of geometry and material properties such as hardness and Young’s modulus. The material properties of the polisher and workpiece were set following the descriptions in Section 2. The slurry was not involved in the model. Figure 2a,b shows the simulated cross-sectional view of stress distribution without tool wear at 20 mN and stress distribution on the workpiece under different loads, respectively. It is noted that the simulated values in this figure may not be quantitatively accurate due to the difference between the actual and setting values of material properties, but the results are adequate for qualitative comparison. It is found that with the increase of force, the contact area was enlarged, and the center part became flatter which indicated that it will be more difficult for the abrasives to be involved in the polished area. Figure 3a,b shows the cross-sectional view of stress distribution with wear diameter of 100 µm and stress distribution on the workpiece with different wear diameters, respectively. According to the results, it can predict that the stress at the central part of contact area was less than the edge, so less material will be removed from central part and ideal tool influence function cannot be obtained.

## 4. Results and Discussions

### 4.1. Effect of Vibrating Motion

**Figure 4:**
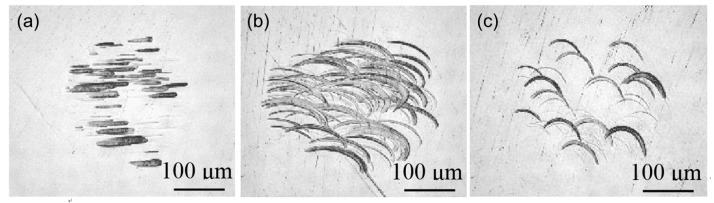
Observation of vibration traces under (**a**) lateral, (**b**) circular and (**c**) elliptical vibrating motions.

Firstly, the effect of different vibration traces on the workpiece surface was observed using a microscope. The magnetostrictive vibrating polisher vibrated at a fixed position on the workpiece surface under lateral, circular and elliptical vibrating motion respectively according to the conditions in Table 1 for a few seconds at 50 mN using a grain size of 1 μm diamond slurry. The results are shown in Figure 4. The granules in diamond slurries were driven by the vibration performing two-body abrasion and three-body abrasion functions to remove material, so the movement of them follows the vibration traces of the polisher. The scratches showed how the material removal process happened on the workpiece surface under different vibrating motions, and the obvious ones were mainly generated by the two-body abrasion when abrasives became embedded and slide over the surface of the workpiece under a relatively large polishing force. Compared with the scratches on the lapped surface, scratches generated by vibration were much shorter (under 100 μm), so a better surface finish could be obtained. Also, during the polishing process, as the polisher scans on the workpiece surface, the scratch effect will be minimized.

**Figure 5:**
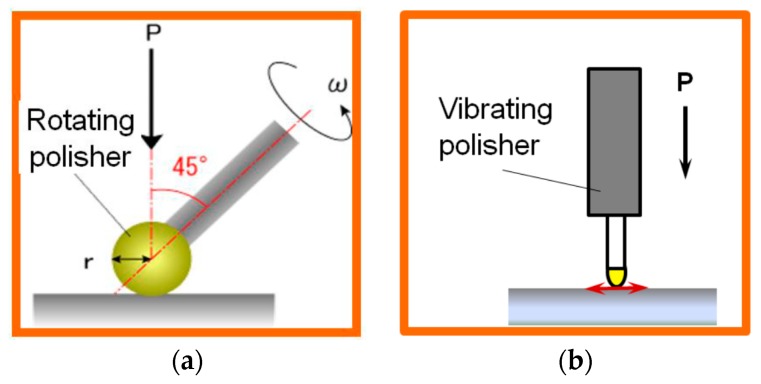
Mathematical models of polishing using (**a**) rotating and (**b**) vibrating tools.

A comparison between rotational polishing and vibration-assisted polishing is shown in Figure 5. For rotational polishing, the polishing speed which is represented by the relative velocity between polisher and workpiece *V* is given by:

(1) $$V=\sqrt{2}\pi rw$$

where, *r* is the radius of the polisher and *w* is the rotation speed. For vibration-assisted polishing, in the case of lateral vibrating motion, the average value is adopted for ease of comparison and *V* is expressed as:

(2) V=4λv

where *λ* and *v* are the amplitude and frequency of vibration respectively, while in the case of elliptical vibrating motion, *V* is approximately changed to:

(3) V=2πbv+4(a−b)v

where *a* and *b* are the long axis and minor axis of elliptical vibration, respectively. In the case of circular vibrating motion, *a* equals *b*, so *V* is changed to:

(4) V=2πrv

where *r* is the radius of circular vibration. So, the circular vibrating motion will generate a higher removal rate than a lateral vibrating motion with a gain of *π*/2. It is found that in the case of rotational polishing, the relative velocity has a linear relationship with the radius of the polisher, while in vibration-assisted polishing the relative velocity is independent of the radius of the polisher, demonstrating that the material removal efficiency is not affected by the size of the polisher.

**Figure 6:**
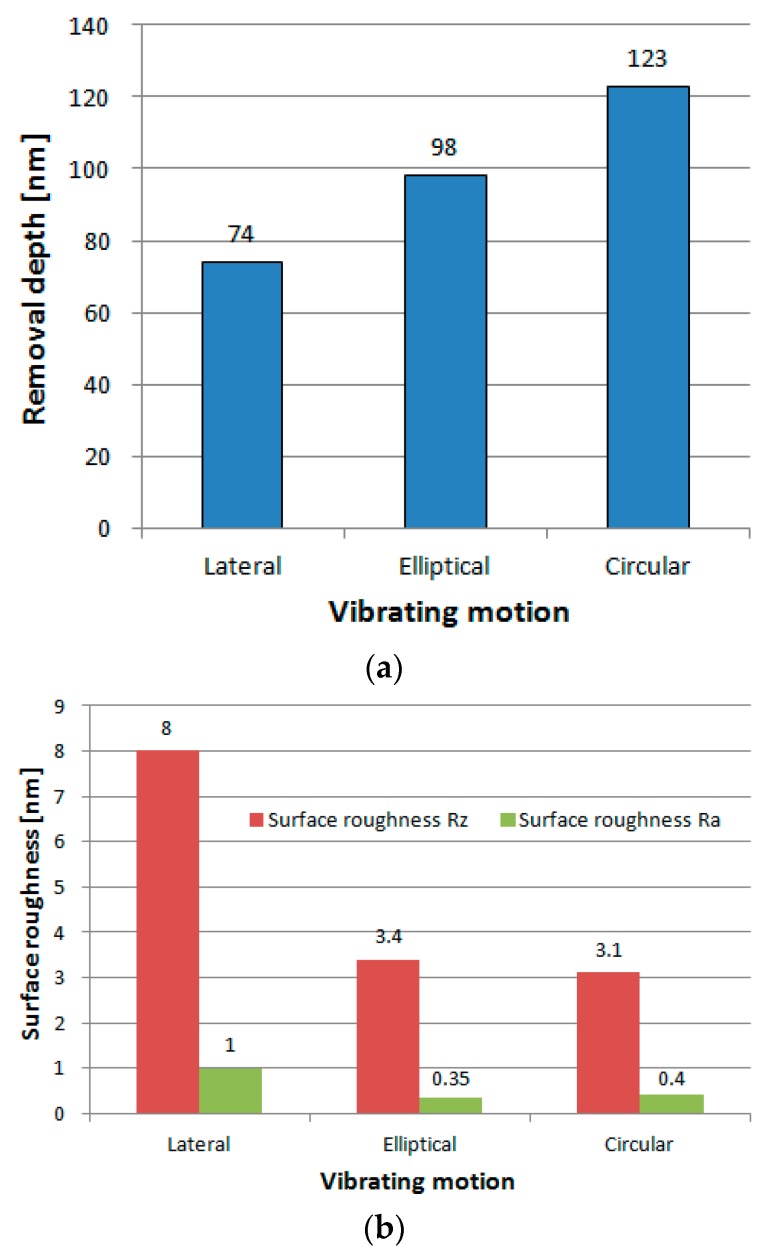
Effects of vibrating motions on (**a**) material removal and (**b**) surface roughness.

**Figure 7:**
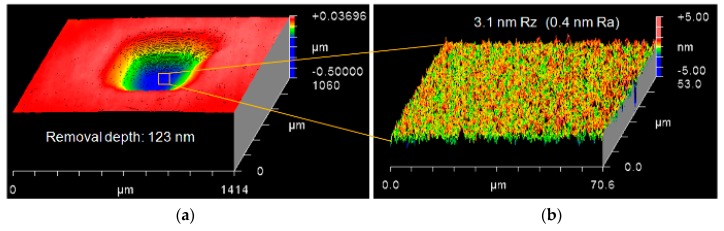
(**a**) Material removal depth and (**b**) surface roughness by circular vibrating motion.

Further investigations were conducted to compare material removal depth and surface roughness under different vibrating motions according to the conditions in Table 1. The magnetostrictive vibrating polisher scanned a raster path on the workpiece for one cycle. According to Preston’s equation [25], the removal depth *δ* should be proportional to the average value of *V* when other parameters are fixed. The polishing force of 5 mN and the grain size of diamond slurry of 0.1 μm is adopted. The results are summarized in Figure 6. This shows the average value of experiments in 10 times with measurement size of surface roughness of 70 μm × 50 μm. It is found that circular vibrating motion has the highest removal depth up to 123 nm and the gain of circular vibrating motion by lateral vibrating motion shows an agreement with the theoretical value. The surface roughness is reduced over 50% by the 2D vibrating motion (elliptical and circular) than that of the 1D (lateral) vibrating motion. Figure 7 shows the measurement data by circular vibrating motion.

### 4.2. Effect of Abrasives

**Figure 8:**
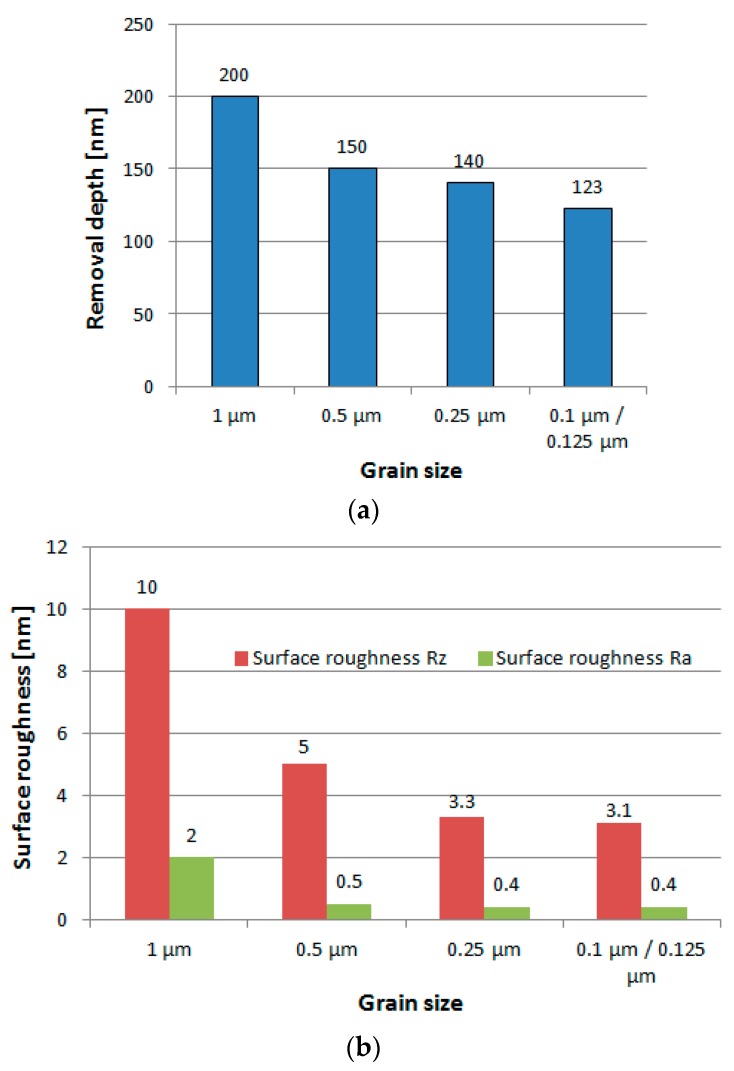
Effects of abrasive grain size on (**a**) material removal and (**b**) surface roughness.

The polishing characteristics of diamond slurries with different grain size were compared under circular vibrating motion with a polishing force of 5 mN. The grains of diamond slurries which have sharp edges demonstrated that the material removal process was mainly performed in nano- and subnano-cutting. The experiment results (see Figure 8) show that both removal depth and surface roughness decrease when grain size of diamond slurry becomes smaller. For practical application in micro-optic mold finishing, the grain size of 0.5 μm and 0.25 μm will be more suitable concerning both removal depth and surface roughness.

### 4.3. Effect of Pressure Conditions

**Figure 9:**
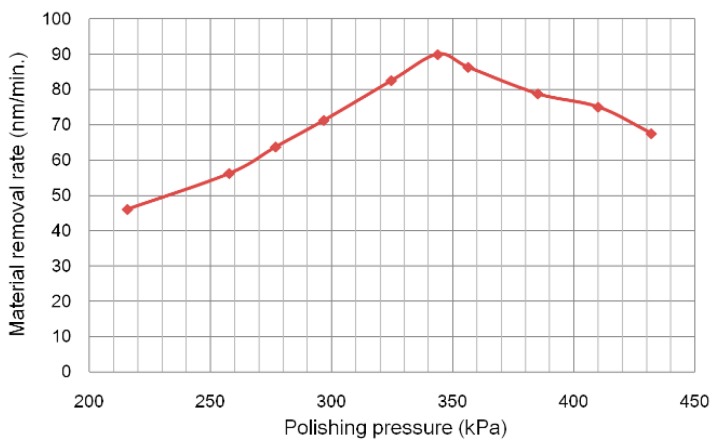
Material removal rate (MRR) change as a function of polishing pressure.

Experiments were conducted to examine the relationship between polishing pressure and MRR. Circular vibrating motion with conditions shown in Table and diamond slurry with grain size of 0.1 μm of were adopted. The average polishing pressure was obtained by a polishing force dividing the contact area. The results are shown in Figure 9. It is found that the MRR showed agreement with Preston’s equation when the polishing pressure was under 345 kPa. But when the polishing pressure exceeded 345 kPa, the removal rate decreased gradually with the increase of polishing pressure.

**Figure 10:**
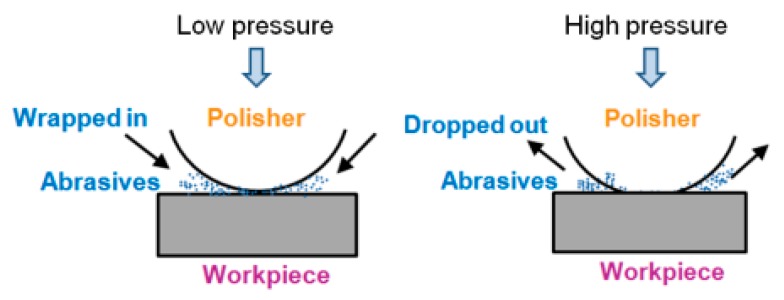
A general model of the material removal mechanism in vibration-assisted polishing.

To explain this phenomenon, a model of material removal in general for micro-optic mold polishing is presented in Figure 10. Although the physical scale of material removal processes in polishing is difficult (practically impossible) to observe directly, there is a general view that there are two kinds of abrasive motion, which are two-body abrasion and the three-body abrasion effect, on the workpiece during loose abrasive polishing process. Two-body abrasion happens when abrasives become embedded and slide over the surface, while three-body abrasion is generated when abrasives become freely rolling abrasives [20]. In this experiment, since the urethane polisher is very soft and the polishing spot is very small (under 0.2 mm^2^), it is easy to generate a high polishing pressure. When the polishing pressure is under 345 kPa, the concentration of granules in diamond slurry between polisher and workpiece keeps constant, and the MMR increases with the increase of polishing pressure although some two-body abrasives transform to the three-body abrasives. However, when the polishing pressure is over 345 kPa, due to the high pressure, some of two-body abrasives will be dropped out from the contact area of the polisher and workpiece while some of them will transform to the three-body abrasives, so the total number of acting granules reduces and the MRR decreases.

### 4.4. Effect of Tool Wear

The aforementioned experiments were conducted under the conditions of no significant tool wear. However, during the polishing process, wear of the polisher occurs while the material is removed, and the MRR will be changed as wear persists. In vibration-assisted polishing by loose abrasive, the wear rate of the polisher is correlated with vibration amplitude and frequency, material and concentration of slurry and polishing pressure. The major fabrication process of the urethane polisher was followed by Phenol resin gluing, cutting or grinding and urethane-coating. During the polishing process, the polisher wears on the top of the head where it contacts with the workpiece, and the head becomes flat gradually. 

**Figure 11:**
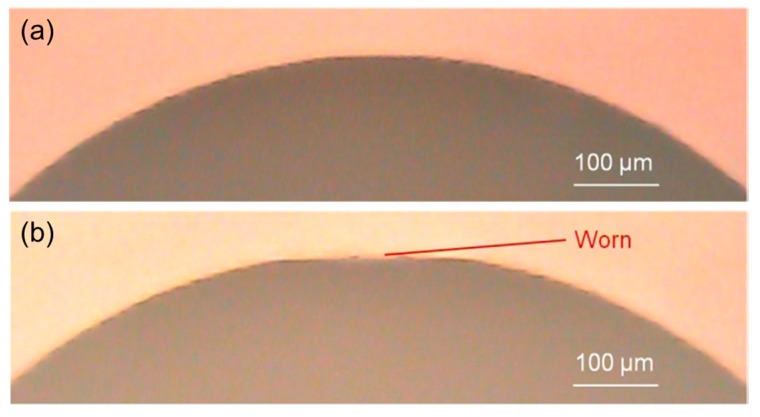
Shape changes on the polisher’s head (**a**) before polishing and (**b**) after polishing.

**Figure 12:**
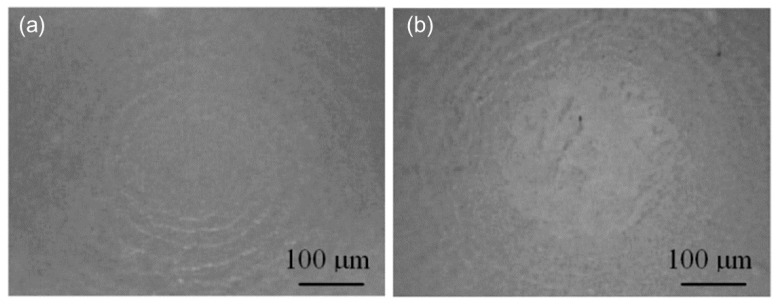
Surface changes on the polisher’s head (**a**) before polishing and (**b**) after polishing.

**Figure 13:**
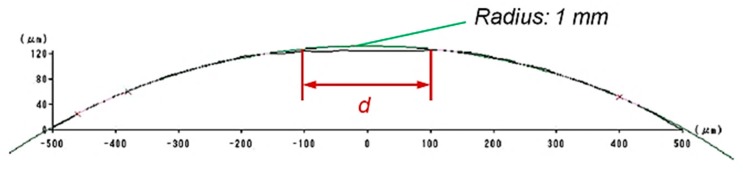
Wear diameter *d*.

Since the wear volume of the polisher is quite difficult to measure precisely, as an alternative method the wear diameter on the head of the polisher was tested. Figure 11 shows shape change on the polisher’s head before and after polishing, and Figure 12 depicts the surface changes on top of the polisher’s head before and after polishing by a microscope. A measurement result is shown in Figure 13. The wear diameter of the polisher is defined as *d* for the following investigations.

**Figure 14:**
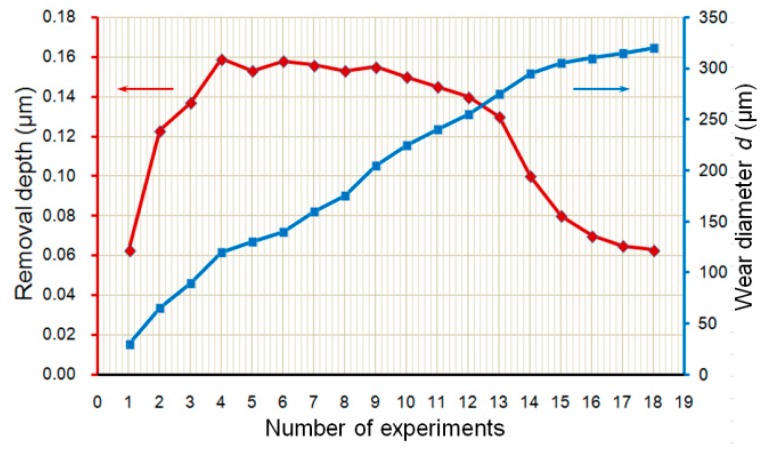
Relations of the polisher’s wear and material removal depth.

To evaluate the effects of polisher’s wear on material removal depth, some polishing experiments were conducted. The polisher scanned a raster scanning path and vibrated in a circular vibrating motion under a constant pressure of 10 mN on the surface of the workpiece. The experiment with the same conditions was repeated 11 times with the change of polishing areas on the workpiece surface. According to the experimental results in Figure 14, it is found that the wear process was almost divided into three steps. During the 1st step (*d* < 50 μm), both wear diameter and material removal depth increased, and the material removal depth goes to a maximum. At the 2nd step (50 μm ≤ *d* ≤ 300 μm), the polisher wears proportional to the number of experiments, but the material removal depth keeps almost constant just with a little decrease. From the 3rd step (*d* > 300 μm), the polisher’s wear speed reduced while the material removal depth decreased dramatically and after that showed a tendency to a constant value.

**Figure 15:**
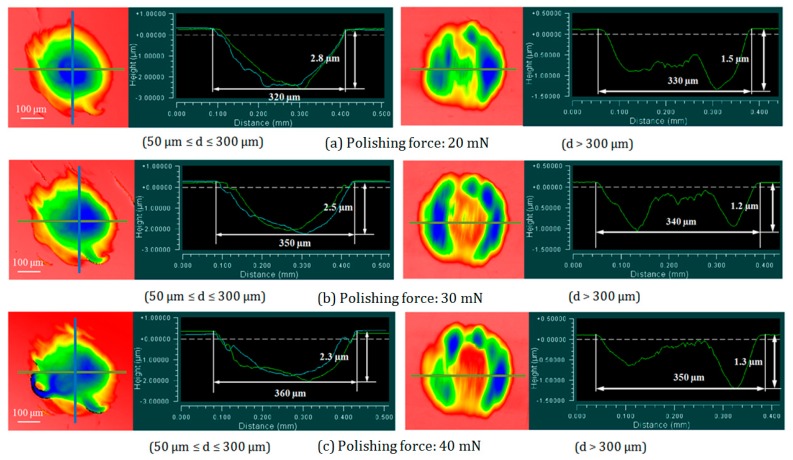
Comparison of tool influence functions with 50 μm ≤ *d* ≤ 300 μm and *d* > 300 μm.

The reason why material removal depth reduces when *d* > 300 μm could be explained through observing the shapes of the tool influence function to some extent. Figure 15 shows the comparison of tool influence functions under the conditions of *d* ≤ 300 μm and *d* > 300 μm at a polishing force of 20–40 mN. When *d* ≤ 300 μm, the shape of the removal function was center-concave and symmetrical which is near to the ideal model of removal function. But when *d* > 300 μm, the acting polishing abrasives became difficult to reach the contact-center of the polisher and workpiece, so the material in the center of the removal function was left and the removal depth reduced. The result also indicated that with the increase of polishing force, the center depth of the tool influence function decreased which led to a low MRR.

## 5. Conclusions

In this paper, some insights into the material removal mechanism in vibration-assisted polishing were revealed through investigating the effects of process parameters on material removal quantitatively. According to the experimental results, some conclusions can be obtained as follows:

(1) 2D vibrating motion created a better surface roughness with higher removal efficiency than 1D vibration motion. The smaller the grain size of the diamond slurry, the better the surface roughness, but the MRR was reduced.
(2) MRR increased when the polishing pressure was under 345 kPa. However, when the polishing pressure exceeded 345 kPa, MRR decreased gradually with the increase of polishing pressure.
(3) MRR was greatly affected by the wear of the polisher when wear diameter on polisher’s head exceeded 300 μm.

In summary, the polishing pressure condition which affects the concentration of granules in diamond slurry and transitions between two-body abrasives and three-body abrasives plays a key role in revealing insights into the material removal mechanism. Future work will be continued on the effect of polishing pressure conditions on the surface roughness to further investigate the material removal mechanism.

## Figures and Tables

**Table 1:** Main polishing conditions.

Workpiece Material	Tungsten Carbide
Polisher head Radius Hardness	Polyurethane 1 mm IRHD 90
Abrasive Grain size Density	Diamond slurry 0.1 μm/0.125 μm/0.25μm/0.5 μm/1 μm 1 wt %
Polishing force	*W* = 5–50 mN (Increment: 5 mN)
Vibrating motion	Lateral (*λ* = 43 μm) Circular (*r* = 30 μm) Elliptical (*a* = 40 μm, *b* = 16 μm)
Frequency	9.2 kHz
Polishing scope Pitch size Scanning speed	400 μm × 400 μm 20 μm 3.5 mm/min
